# Open science must include effective results dissemination to study participants

**DOI:** 10.1371/journal.pmed.1004780

**Published:** 2025-10-15

**Authors:** Ka Hin Tai, Gérard Legoff, Anne Le Louarn, Nchangwi Syntia Munung, Florian Naudet

**Affiliations:** 1 Univ Rennes, Inserm, EHESP, Irset (Institut de recherche en santé, environnement et travail)—UMR_S 1085, Rennes, France; 2 France Rein Bretagne, Laillé, France; 3 GCS CNCR (Comité National de Coordination de la Recherche), Paris, France; 4 Division of Human Genetics, Faculty of Health Sciences, University of Cape Town, South Africa; 5 Institut Universitaire de France (IUF), Paris, France; 6 Université Rennes, CHU Rennes, Inserm, Centre d’Investigation Clinique de Rennes (CIC1414), Service de Pharmacologie Clinique, Institut de Recherche en Santé, Environnement et Travail (Irset), UMR S 1085, EHESP, Rennes, France

## Abstract

Open science often centers around publications and data transparency. This Perspective discusses how and why disseminating results to study participants is essential for maximizing the values and benefits of open science.

## Teaser

Open science often centers around publications and data transparency. We highlight how and why disseminating results to study participants is essential for maximizing the values and benefits of open science.

Science is, and should always be, a public good. UNESCO defines open science as making scientific knowledge openly available, accessible, and reusable for everyone [1]. Effectively and transparently disseminating research findings is therefore a cornerstone of open science. Yet dissemination is often equated to open publications or data, overlooking other stakeholders: study participants. Making results accessible to participants respects them as contributors and co-producers of knowledge [2]. While not all participants may wish to receive results, the opportunity should always exist in a simple, accessible form.

A recent PLOS Medicine study [3] reviewed 96 studies on global practices, expectations, barriers, and impacts of sharing research results with study participants. The results showed that most participants expect results regardless of outcome, seeing this as recognition of their contribution and a foundation for trust. Researchers described dissemination as a moral obligation, but practices remain inconsistent: although two-thirds of surveyed U.S. researchers thought results should always be shared, only 8% had concrete plans to do so. Common methods included mailed lay summaries or letters, while group presentations and workshops were more often reported in lower-income or community-based settings such as Uganda and Brazil.

Here, we focus on clinical trials to illustrate these issues, but the principles—respect for participants, transparent reporting, and meaningful involvement—apply equally across health research.

## Trials begin with participants

Clinical trials are meant to be patient-centered, designed to generate medical knowledge for public welfare. Some argue that as trials are initiated and funded by sponsors, their outputs are private property. This view ignores the fact that no research can proceed without participants’ involvement. Yet it remains common to see participants framed as passive data points [4], rather than as partners in the scientific enterprise.

Approaching clinical trials as a collective enterprise for public benefit demands a shift in mindset. Researchers become stewards, not proprietors, of shared knowledge. Only through meaningful involvement of patients and the public can trials be ethically justified and socially impactful; by mandating transparency, enabling equitable access, and valuing inclusive participation, it realigns research practice with its public purpose.

## Extending open science to participants

Engagement begins with information. Before the trial, participants must be clearly informed about the study’s purpose, risks, potential benefits, and their legal rights—now a legal standard ensuring autonomy and protection. After the trial, results should be shared in plain language and through appropriate channels. Transparent reporting, regardless of whether findings are positive, negative, or unexpected, reinforces the credibility of research and meets societal expectations. Initiatives like AllTrials [5], a global campaign calling for the registration and reporting of all clinical trials, and TranspariMED [6], an advocacy group that works to end evidence distortion by pushing for rapid public disclosure of all clinical trial results, have mobilized global support and helped recover thousands of trial results that would otherwise have been lost.

Even when researchers wish to share results, practical barriers often stand in the way [3]. From the review, more than half respondents in one study reported financial constraints that hampered dissemination, while two-thirds of health researchers in another study reported difficulty in dissemination because of missing contacts or remote settings. The review found no consensus on best practices, and while existing frameworks to guide result dissemination are “now in early development,” they have remained invalidated across diverse settings, populations, or clinical domains [3]. Dissemination also requires time, dedicated resources, and institutional support, which are often lacking. Training in how to prepare lay summaries or communicate sensitive or distressing results was often limited [3]. These are crucial skills when findings may provoke anxiety, disappointment, or even grief. Without adequate funding, guidance, and capacity-building, dissemination risks being treated as an unfunded add-on rather than a core responsibility of ethical research (see Box 1).

**Box 1. When science is not open.**

A controversial study on dyslexia published in 2017 bore concerns on its methodological rigor and reproducibility [7]. Nevertheless, it was used as a rationale to develop and test devices such as flickering glasses and lamps in subsequent trials. The sponsors of the two trials allegedly withheld data by preventing publication, and, more specifically, the sponsor of the glasses trials reportedly failed to return results to both participants and investigators [8]. However, throughout the years, the two patented inventions building upon the trial reached the market without evidential benefit. This lack of transparency, preventable with a simple yet due returning of results to participants and the public, undermines scientific accountability and erodes public trust. More critically, it leaves patients exposed to interventions with unproven efficacy, sometimes even marketed as evidence-based solutions. Despite existing regulations and ethical guidelines, result reporting remains poorly enforced, allowing sponsors to bypass scrutiny with little consequence. In the end, the absence of transparency does not just hinder science, it actively disservices the very individuals research is meant to help.

In stark contrast, the RECOVERY trial during the COVID-19 pandemic exemplifies how rapid and transparent dissemination can directly benefit participants, science, and society. From the outset, the trial website provided consent forms, plain-language updates, and videos explaining the main results, ensuring accessibility for participants and the public. Within months of initiation, the trial demonstrated that dexamethasone reduced mortality in severely ill patients [9]. Instead of waiting for formal publication, the team immediately shared results through press briefings, supported by a publicly available protocol. This swift, although controversial, communication enabled the UK’s National Health Service to update treatment guidelines within hours, saving hundreds of lives [10]. A detailed preprint followed soon after.

A second level is involvement. The public can be consulted in trial design, result interpretation, and even co-develop research protocols—extending into citizen science. Patients bring lived experience that can highlight what outcomes matter most and how interventions are perceived. Such collaboration enhances trial relevance, feasibility, and public acceptance [11]. Research demands a clear alignment of study inquiries with patient needs. Without patient and public involvement, trials risk inadequate participant recruitment, suboptimal study design, and unresponsiveness to patients’ experiences and feedback [12], producing less meaningful outcome and wasting resources.

## Open science practices must be part of IRB assessments

Institutional Review Boards (IRBs) are essential in maintaining the integrity of study processes and serve as custodians of participant trust. Scientific trustworthiness comes from methodological rigor, verifiability, and transparency, whereas research ethics is grounded in protecting human rights, dignity, and welfare. However, as outlined in the Declaration of Helsinki, ethical obligations extend further to include scientific integrity, encompassing the dissemination of results to both trial participants and the public.

To keep up with the evolving expectations, open science practices should also be systematically reviewed and explicitly encouraged by IRBs, including open access publications, responsible data sharing, and dissemination of findings to participants and the public. For example, the WHO’s recommended protocol format already requires a section on dissemination, covering scientific publication and communication with participants, communities, and policy-makers. This shows such requirements are feasible and ethically recognized. IRBs could similarly require trial protocols to include concrete dissemination plans outlining how and when results will be returned to participants, in what format, and what resources or training are allocated for this. The boards could then assess whether these plans are realistic, ethically sound, and culturally appropriate, just as they currently review informed consent procedures. Embedding such requirements would ensure accountability and normalize dissemination as a core ethical duty, addressing the lack of institutional guidance and consistency highlighted in the systematic review [3].

## A final message

Open science seeks to break down barriers, foster transparency, and ensure that the benefits of research are shared broadly, especially including those who participated in making it happen. Returning results to participants is a cornerstone of ethical and open research, where IRBs play a key role in ensuring that such practices are implemented correctly and evaluated adequately. Inclusivity of the public balances out the current skewed power dynamics in science, leading to health democracy. After all, trials are done for patients, but they all begin from patients.

## Abbreviation

**:**
IRBs: Institutional Review Boards

## References

[1] UNESCO. UNESCO Recommendation on Open Science [Internet]; 2021 [cited 2025 Aug 3]. Available from: https://unesdoc.unesco.org/ark:/48223/pf0000379949

[2] South A, Bale C, Chowdhury E, Churchill A, Donoghue K, Grieveson S, et al. Sharing clinical trial results with participants: important, expected and possible. Trials. 2025;26(1). 10.1186/s13063-025-08802-0 PMC11948716 40140937

[3] Bagita-Vangana M, Unger HW, Thriemer K. Current global practice and implications for future research on disseminating health research results to study participants: a systematic review. PubMed. 2025;22(8):e1004569–9. 10.1371/journal.pmed.1004569 PMC12352677 40811427

[4] Beier K, Schweda M, Schicktanz S. Taking patient involvement seriously: a critical ethical analysis of participatory approaches in data-intensive medical research. BMC Med Inform Decis Mak. 2019;19(1). 10.1186/s12911-019-0799-7 PMC6482526 31023321

[5] Sense About Science. The AllTrials campaign [Internet]. London: Sense About Science [cited 2025 Aug 3]. Available from: https://www.alltrials.net/find-out-more/why-this-matters/the-alltrials-campaign/

[6] TranspariMED. TranspariMED [Internet]. TranspariMED; 2024 [cited 2025 Aug 3]. Available from: https://www.transparimed.org/

[7] Naudet F, Seidenberg M, Dorothy D. Comment on Le Floch & Ropars (2017) “Left–right asymmetry of the Maxwell spot centroids in adults without and with dyslexia.”. Proc Biol Sci. 2024;291(2017). 10.1098/rspb.2023.2060 PMC10898962 38412972

[8] Borrell B. Controversial dyslexia study marred by methodological and ethical problems, researchers say. The Transmitter [Internet]; 2024 Feb 27 [cited 2025 Jul 20]. Available from: https://www.thetransmitter.org/ethics/controversial-dyslexia-study-marred-by-methodological-and-ethical-problems-researchers-say/

[9] RECOVERY Collaborative Group. Dexamethasone in hospitalized patients with covid-19—preliminary report. N Engl J Med. 2020;384(8):693–704. 32678530 10.1056/NEJMoa2021436 PMC7383595

[10] Knowlson C, Torgerson DJ. Effects of rapid recruitment and dissemination on Covid-19 mortality: the RECOVERY trial. F1000Res. 2020;9:1017. doi: 10.12688/f1000research.25842.2 33447376 PMC7783530

[11] Gobat N, Slack C, Hannah S, Salzwedel J, Bladon G, Burgos JG, et al. Better engagement, better evidence: working in partnership with patients, the public, and communities in clinical trials with involvement and good participatory practice. Lancet Glob Health. 2025;13(4):e716–31. doi: 10.1016/S2214-109X(24)00521-7 40155109

[12] Pijuan J, Palau F. Patient involvement in clinical trials: a paradigm shift in research. Orphanet J Rare Dis. 2025;20(1). 10.1186/s13023-025-03573-y PMC11812247 39930424

